# TRAINING ASIAN AND WHITE FAMILY CAREGIVERS LIFE REVIEW DEPRESSION INTERVENTION: A MIXED-METHODS COMPARATIVE STUDY

**DOI:** 10.1093/geroni/igae098.2856

**Published:** 2024-12-31

**Authors:** Christina Miyawaki, Angela McClellan, Erin Bouldin

**Affiliations:** University of Houston, Houston, Texas, United States; University of Houston, Houston, Texas, United States; University of Utah, Salt Lake City, Utah, United States

## Abstract

Approximately 10% of older Americans living with Alzheimer’s dementia experience depression, yet behavioral interventions remain scarce. We developed a 6-week depression intervention, Caregiver-Provided Life Review (C-PLR) based on life review therapy. The purposes of this study were to examine the feasibility of training Asian and White family caregivers in life review skills and examine the impact on care recipients’ depressive symptoms as 77% of Asian dyads conducted the study in their native language. Using a mixed-methods design, Asian (n=15: 7=Chinese, 4=Japanese, 2=Vietnamese, 1=Filipino and Korean) and White (n=25) caregiver-care recipient dyads were trained. We measured care recipients’ pre- and post-intervention depression (primary outcome), life satisfaction, caregivers’ burden, rewards of caregiving, and dyads’ relationship quality (secondary outcomes) scores, and compared them by paired t-tests. We evaluated the feasibility of caregivers’ training by fidelity check-in scores. All dyads completed the study. Asian (mean age=52 years) and White (60 years) caregivers were working, female, and in good/excellent health. Their care recipients (Asian=82 and White=80 years) were female and in poor/fair health. Both Asian and White care recipients’ depression and dyads’ relationship from baseline to post-intervention significantly improved (Asian=1.9, p< 0.001; 0.9, p=0.029; White=1.0, p=0.034; 0.6, p=0.033, respectively). Both caregivers’ fidelity scores were identical and similarly high (15.4; 15.3, p=0.91). Qualitative interviews of both caregivers supported the sentiments of the quantitative results. The language of Asian dyads appeared no influence on any outcomes. This study demonstrates that the C-PLR can be added to a list of successful non-pharmaceutical interventions for Asian and White older adults.

